# Overcoming Catastrophic Interference in Connectionist Networks Using Gram-Schmidt Orthogonalization

**DOI:** 10.1371/journal.pone.0105619

**Published:** 2014-09-02

**Authors:** Vipin Srivastava, Suchitra Sampath, David J. Parker

**Affiliations:** 1 School of Physics, University of Hyderabad, Hyderabad, India; 2 Centre for Neural and Cognitive Sciences, University of Hyderabad, Hyderabad, India; 3 Department of Physiology, Development and Neuroscience, University of Cambridge, Cambridge, United Kingdom; Tokai University, Japan

## Abstract

Connectionist models of memory storage have been studied for many years, and aim to provide insight into potential mechanisms of memory storage by the brain. A problem faced by these systems is that as the number of items to be stored increases across a finite set of neurons/synapses, the cumulative changes in synaptic weight eventually lead to a sudden and dramatic loss of the stored information (catastrophic interference, CI) as the previous changes in synaptic weight are effectively lost. This effect does not occur in the brain, where information loss is gradual. Various attempts have been made to overcome the effects of CI, but these generally use schemes that impose restrictions on the system or its inputs rather than allowing the system to intrinsically cope with increasing storage demands. We show here that catastrophic interference occurs as a result of interference among patterns that lead to catastrophic effects when the number of patterns stored exceeds a critical limit. However, when Gram-Schmidt orthogonalization is combined with the Hebb-Hopfield model, the model attains the ability to eliminate CI. This approach differs from previous orthogonalisation schemes used in connectionist networks which essentially reflect sparse coding of the input. Here CI is avoided in a network of a fixed size without setting limits on the rate or number of patterns encoded, and without separating encoding and retrieval, thus offering the advantage of allowing associations between incoming and stored patterns.

PACS Nos.: 87.10.+e, 87.18.Bb, 87.18.Sn, 87.19.La

## Introduction

Nervous systems have two basic requirements: they must be stable and thus able to generate reliable specific outputs, while at the same time they must be flexible to allow the output to change during development or as a result of experience. This is the “stability-plasticity dilemma” [1], and it is a concern to both neurobiologists who want to understand how nervous systems cope with constantly changing internal and external conditions, and those working on artificial neural networks. While not exclusively related to it, this problem is often considered in relation to memory. The analysis of memory systems has been a major focus of neuroscience research, but there are still many unanswered questions that need to be addressed at both the experimental and theoretical levels. In terms of the stability-plasticity problem, the question is how a system can store new input patterns across shared components without disturbing previously stored information in those components.

One of the first considerations of this problem was highlighted by Bienenstock, Cooper and Munro [2], who suggested that long-term potentiation (LTP), a proposed mechanism for learning and memory [3], could suffer from an inherent instability (the BCM model). They suggested that in systems with a set threshold for plasticity the potentiation of a synapse by a particular input that exceeded the threshold could leave that synapse open to further potentiation when another, non-salient, input was presented (this has also been referred to as the “ongoing plasticity” problem; see [4]). Due to the initial potentiation of the synapse, non-salient or random inputs caused by a non-stationary environment could exceed the threshold for plasticity, resulting in the potential for run-away cycles of potentiation which would alter the synaptic changes associated with the original memory. This would effectively overwrite the original memory, and in biological systems if left unchecked, excessive activation could also lead to epileptogenic or excitotoxic damage and cell death [5]. The opposite effect could occur with long-term depression, where a synapse is weakened when the input falls below a depression threshold: in this case there could be a positive feedback loop that results in the successive depression of the synapse.

While the exact relationship is not clear, a similar effect may occur in artificial neural networks. When the number of sequentially recorded/stored patterns exceeds a critical value there is a sudden and complete loss of previously stored inputs [6]. This example of retroactive interference is called catastrophic interference (CI) and is caused by the sharing of connections whose weights are changed by the presentation of specific inputs. As more patterns are stored the weights are changed and beyond a critical point new inputs erase the memory of previous inputs. If the memories happen to be overlapping, or correlated, which essentially means that several of their elements are similar (the mathematical meaning is explained in [7], [8]), then a particular synapse may get increasingly more potentiated (or depressed), thus resembling the stability issues addressed in the BCM model. In human memory, although recently stored or retrieved memories are labile (e.g. [9], [10]), it is rare to find a complete disruption or loss of previously acquired information: a relatively small and gradual reduction (“graceful degradation”) rather than a large catastrophic loss usually occurs (e.g. [11]; but see [12], [13], [14]). That a catastrophic interference like effect can be shown under some conditions is of interest, as it suggests a basic limitation of storage systems that use a finite (although large) number of components, and further that the brain has presumably evolved a way of avoiding this phenomenon, allowing new information to be stored without disrupting previously stored information (but see [15]). Understanding this capability of the brain and how it can be applied in artificial networks could be of interest to both the psychological/neurobiological and technological communities.

Various strategies have been suggested to overcome the effects of CI. These include the separation of new inputs from those previously stored by using a cascade of synaptic states [16]; separate encoding and storage systems (e.g. hippocampal and neocortical networks; [17]); setting limits on the magnitude or rate of learning [18]); the creation of new storage components through neurogenesis [19]; anti-Hebbian plasticity [20]; reducing the overlap between different patterns by sparse coding or by limiting or “sharpening” the number of units used to encode an input, orthogonal recoding of inputs, or interleaving, refreshing previously stored inputs with the new patterns to be learnt (see [12] for review by French and also Guyon et al. for an orthogonalization like approach that involves pseudoinverse of state matrix). Connectionist architectures use interleaving algorithms that require the network to repeatedly cycle through the patterns to be learned; after the entire set of patterns has been presented many times, the network is expected to converge on an appropriate set of weights for the complete set. The problem of CI has also been addressed by curbing the growth of synaptic efficacy by putting bounds on plasticity (see [4]). This is biologically realistic, as it reflects “soft-bound” plasticity, the difficulty of potentiating synapses that are initially strong [21]. While these approaches can overcome effects in theoretical analyses, they all have limitations in terms of their implementation or their biological relevance [22], [23].

The potential parallels between the stability issues in biological and artificial systems inspire us to study the run-away cycle of potentiation using strategies employed to overcome CI. The BCM model suggested a form of self-organising or homeostatic plasticity that could preserve function within set limits while still offering the possibility of directed plastic changes through a sliding plasticity threshold [24], [25]. This threshold would be increased after LTP (or decreased after long-term depression, LTD) to ensure that the potentiation (or depression) needed to encode relevant changes could occur, but further potentiation would not occur with non-salient or random ongoing inputs, only when the new input exceeded the new plasticity threshold [26], [24]. In this case the plasticity of the synapse would be dependent on the previous activity of the synapse, an example of metaplasticity [27].

The BCM model is an attractive and biologically plausible proposition for introducing bounds on synaptic plasticity that could help to overcome the stability-plasticity dilemma. However, as with most attempts to relate cellular and synaptic effects to network function (e.g. memory), while there is evidence for a shifting plasticity threshold the extent to which a BCM-like effect is involved in human memory has not been established, and the model has not been considered in artificial systems in the context of catastrophic interference. We show that when Gram-Schmidt orthogonalization is combined with the Hebb-Hopfield model, the model automatically checks the possibility of a run-away potentiation cycle from being set up, and thus attains the ability to eliminate CI.

The model we use is extremely simplified and uses the bare minimum core features of the neural system we wish to study, and its underlying conditions. Consequently it may appear to be far removed from biology. However, it is analytically tractable and is very widely used in theoretical analyses, and it has an inherent property of encoding synapse-like elements that should give the essential science behind the phenomena we are interested in. Also it should generalize to more realistic models, assuming that certain assumptions are met (see Discussion). We believe that the insight we obtain from it may represent real phenomena. Because of the mathematical nature of the model, it is open in that it can, in principle, be generalized indefinitely to include realistic features. At every stage of its generalization (or expansion) to include a new realistic feature, its mathematical tractability has to be ascertained, and in principle the numbers that come out of solving the improved model should be comparable to experimental measurements.

## Inherent Bounds on Post-Synaptic Response in Hopfield Model

### Outline of the model

For mathematical convenience and in line with most connectionist modeling we will consider a fully connected network in which each neuron is connected to all other neurons, and an information is spread over the entire network and stored as changes in synaptic efficacy that depend on the activities of the pre- and the post-synaptic neurons. The same set of neurons and synapses are involved in storage as well as retrieval of information. A neuron is treated as a binary entity, which assumes values +1 and −1 depending on whether it ‘fires’ or ‘does not fire’. An information that comes to be recorded in the network is assumed to trigger ‘firing’ and ‘not firing’ activities among the neurons in an asynchronous manner: the neurons exchange signals (i.e. action potentials) which raise or lower the potentials on post-synaptic neurons, and if the net potential on a neuron exceeds its threshold then it fires (+1), otherwise it remains quiescent (−1). Thus, an information ‘*μ*’ is represented by a vector,
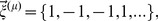
(1)whose components are a collection of +1 and −1 (appearing to be distributed randomly) [28]. The information, represented by a pattern of ±1's spread over the network, is stored in the synapses according to the following learning rule, originally postulated by Cooper [29] to mimic Hebbian synaptic plasticity:
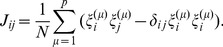
(2)


*J_ij_* is the synaptic efficacy between a pair of neurons *i* and *j*, 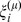 is the *i^th^* component of vector 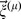, 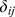 is Kronecker delta function ( = 0 unless *i* = *j*, when it is 1), *N* represents the number of neurons in the network, and *p* is the number of patterns recorded in the network. The right hand side is divided by *N* to normalise the results so that they become independent of the size of the system, i.e. the number of neurons in the network (note that the length of 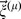  =  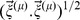  = *N* ^1/2^, so by dividing 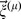, or equivalently each of its components, by *N* ^1/2^ the length of the vector is normalised to *one* regardless of the size of the system). For simplicity we consider *J_ij_* = *J_ji_*, though the model does not impose this restriction, but *J_ii_* = 0 is required for mathematical reasons [30]. The 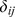 is introduced in the second term on the right hand side to ensure that *J_ii_* = 0. It is assumed that synaptic efficacy between two neurons depends on the activities of the post- and the pre-synaptic neurons, and following Hebb [31], since the efficacy is expected to be high if both neurons fire and low when one of them is not firing, the *J_ij_* is taken as multiplication of 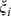 and 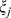. This means that if, for example, the postsynaptic neuron fires independently of the presynaptic neuron the synaptic efficacy will be weakened, which has a correlate in spike timing-dependent plasticity in biological systems (e.g. [32]). However, biologically there is no correlate as to how the efficacy of *J_ij_* can be increased if both the neurons do not fire, as rule (2) would indicate. This rule is referred to as Hebbian learning in spite of the above discrepancy. In practice, the potentiation predicted when neither neuron fires is often ignored by placing a bound on the synapse [33].

Note that the *i*−*j* synapse changes every time a pattern comes to be recorded and the change is added to the changes produced by the previous patterns. Having stored a number of patterns, say *p*, we should test if they are actually stored in the synapses following the Hebbian prescription in (2). We can present one of the *p* learnt patterns to the network and check if it can associate with its original version supposedly embedded in the memory store. The presented pattern, say 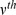, will create local fields on different sites (or neurons) via the synaptic efficacies (or weights) modified in the course of learning *p* patterns as follows,
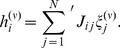
(3)


Here *i* is the post-synaptic neuron, and *j* are the pre-synaptic neurons with respect to *i*. The ‘prime’ on the summation indicates that the sum is over all *j*'s except *i* so that the inputs from all *j* sites add up on *i* and self-connections *J_ii_*'s are excluded. The activity or its absence on pre-synaptic neurons *j* represented by 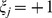 and −1 respectively individually influence the neuron *i* with weights *J_ij_*'s, and these influences (which can be positive or negative since the weights as well as 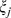 can be positive as well as negative) add up on the post-synaptic neuron *i* to produce a net effect, the local potential *h_i_*. This local field (or potential), which is a measure of total post-synaptic potential (PSP) on neuron *i* can be positive or negative. If its sign matches with the sign of 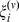, and such agreement happens on the majority of neurons (say, more than 97%, a generally accepted level; see [34] and references therein) then the association is considered to be good and the pattern 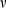 is considered as recalled, or retrieved.

To elaborate it we will substitute for *J_ij_* from eqn.(2). So,
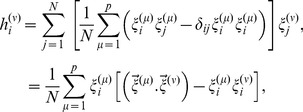
(4)since 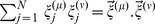, the dot-product of two vectors, and 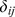 picks out 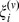 from 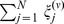 and makes the remaining terms zero; 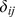 also serves the purpose of ‘the prime’ on 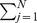, so ‘the prime’ is dropped in eqn.(4). Isolating the 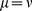 component from 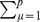 in the first term on the right hand side, we will get *N* from 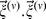 and will be left with 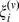. Further, 
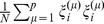
 will give *p*/*N* in either case of 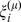 being +1 or −1. Thus, we find that,
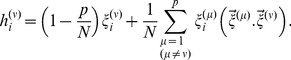
(5)


This rearrangement has enabled us to isolate 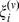, whose sign is to be compared with that of 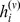, from a jumble of cross terms involving the test pattern ‘
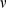
’ and all the other patterns in the memory store represented by ‘*μ*’. This is like separating a signal from a jumbled mixture of cross-talks this signal has with a number of other signals. If 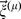
's happen to be mutually orthogonal, the cross-talks will vanish and the memories would work perfectly [30].

### Analysis of post-synaptic potential

**Figure 1:**
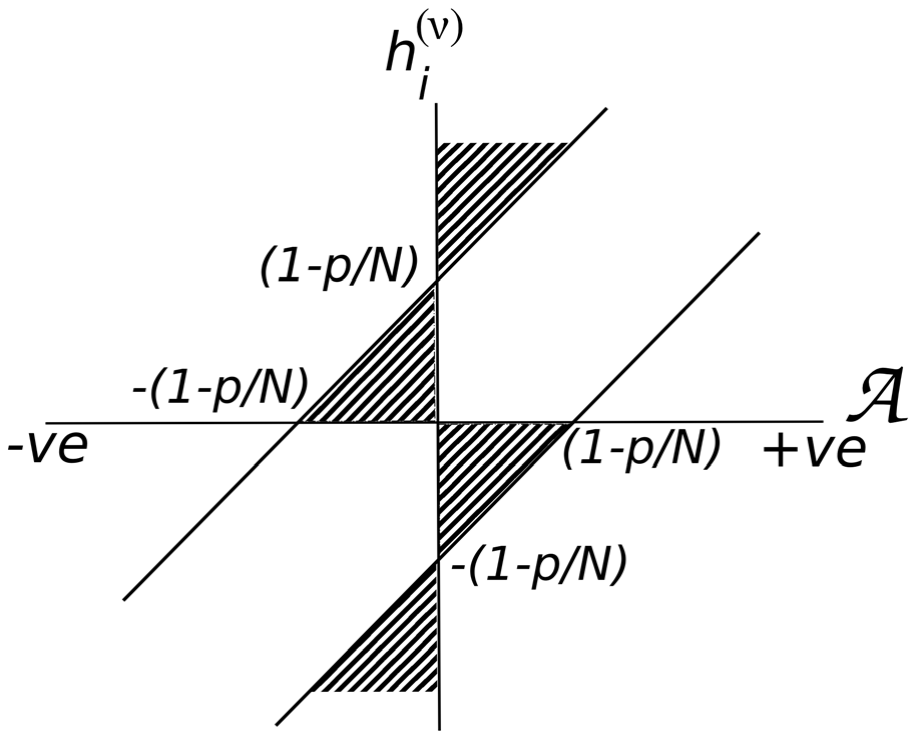
Schematic representation of 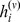, the post-synaptic potential on an arbitrary site *i* when one of the learnt patterns, 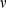 is presented to check for retrieval, versus 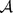, the noise term in eqn.4. The shaded areas represent the domains where 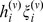 will be positive definite. The bounds on 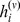 slide up and down with variations in *p* and *N* enabling, at least in principle, plasticity to control CI to some extent.

The sign of 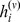 (or PSP) can become unfavourable (i.e. opposite of 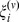) due to the second term in eqn.(5) (let us call it 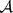). Since the vectors 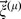 consist of randomly generated +1's and −1's, each of the *p* terms in the second term in the right hand side of eqn (5) will take a fractional value, less than 1, with a random sign (+ or −). Thus, for 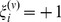, 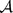 can take any positive or negative value limited by the values of *p* and *N*, but as long as it is greater than −(1−*p*/*N*), 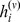 will match in sign with 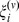. Similarly, for 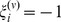, 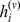 will match in sign with 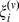 if 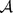 remains less than (1−*p*/*N*). Figure 1 shows the favourable ranges of values of 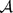 in the form of shaded areas. Note that in general 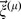
's are not orthogonal to 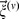. So, the dot products 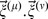 are non-zero. In spite of the signs being randomly + or − the chances of 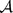 growing arbitrarily large, +*ve* or -*ve*, become increasingly large with increasing *p*. This increases the possibility of CI as explained below.

In eqn.(5) the first term on the right hand side is like signal while 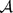 represents noise – note that the first term is obtained by isolating in eqn (4) the relevant component, i.e. *i^th^*, of the pattern being retrieved, i.e. the 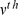 vector, while the overlaps of 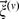 with all the remaining vectors in the memory store are clubbed together in the second term; it is these non-zero overlaps that obfuscate the signal and hence act as noise. From the above we see that as long as the noise 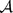 can be bounded by (*p*/*N*−1) from below and by (1−*p*/*N*) from above, 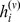 will be confined between (*p*/*N*−1) and (1−*p*/*N*), and CI will be contained. However, as new patterns come to be recorded, there is no intrinsic mechanism in the Hopfield model to control their overlaps with the patterns already in the store and thereby restrict the noise 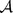 to within the above limits, and thus restrict 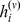 to within the above favourable limits. Thus, as the number of patterns in the store increases the noise builds up and the likelihood of 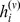 remaining within favourable limits reduces on more of the neurons (*i*'s) in the system and CI becomes inescapable. These bounds on PSP can slide with the variations in *p* and *N*, to make CI more susceptible or less susceptible. If *p* increases (for a given *N*) then the bounds shrink and the system becomes more susceptible to CI, which is understandable since the interference among patterns will increase as their number increases. On the other hand the increasing system size (such that 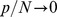) would widen the gap between the bounds and reduce the chances of CI.

Note that outside the above bounds 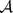 can, in principle, grow to very large positive or negative values, akin to runaway affects in the BCM model (see above). Although indefinitely large positive and negative values of 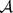 will keep 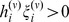 for 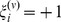 and 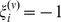 respectively, the fact is that 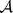 takes positive or negative values in a seemingly uncontrolled and random manner. Therefore, its growth to large values is, in general, detrimental to retrieval (or recall) and leads to CI [34]. This will cause the run-away effect, which will eventually give false (or deceptive) associations with the feature designated by site *i*.

**Figure 2:**
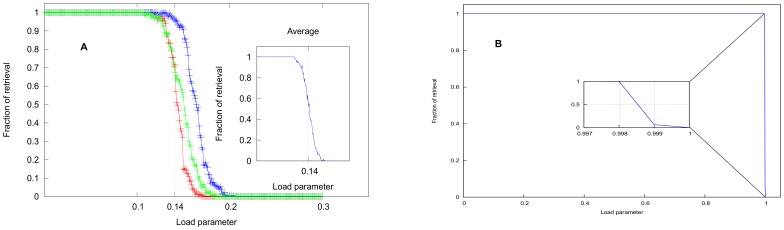
Simulation results for a system of 1000 neurons. (A) Hopfield network showing memory breakdown due to catastrophic interference amongst the stored patterns – the fraction of input patterns that is retrieved drops rapidly around the load parameter, *p*/*N* = 0.14. The results are shown for three sets of patterns and the inset shows the results averaged over 50 sets of patterns. (B) Hopfield network with Gram-Schmidt orthogonalization of the incoming patterns. All the learnt patterns are retrieved perfectly until *p* = *N*, when the retrieval fraction drops to zero abruptly. The inset shows magnification very close to the load parameter  = 1 to highlight the abruptness of the drop. Note that the system does not learn the raw patterns as they are presented but their orthogonalized versions, whereas the retrieval is checked for the raw patterns.

The uncontrolled growth of 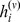 on a large number of sites inevitably leads to catastrophic forgetting in the Hopfield model if the ratio *p*/*N* exceeds 0.14 (see e.g. [30]). In figure 2 we present the result of a simulation showing how degradation sets in in the quality of retrieval as *p*/*N* exceeds 0.14 (details are given in the following section).

## A Way Out of Catastrophic Interference

It is our hypothesis that when a stimulus (or vector) 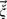 is presented to the system, the system orthogonalizes it with respect to all the vectors in the memory store and then stores the orthogonalized vector 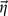 rather than the raw vector 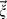 [7]. In real terms this amounts to storing the similarities and differences of the new vector with the old vectors.

Suppose 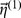, 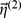, …, 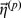 are the orthogonalized versions of 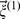, 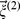, …, 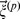, and they are stored in the Hebbian manner as,
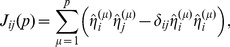
(6)where 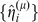 are the components of 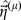 obtained by normalising 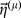 as 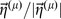. It is not immediately obvious as to how the brain would perform the normalization. While there is physiological and behavioural (e.g. psychophysical) evidence for normalization as a canonical neural computation, its role and underlying mechanisms are still an area of intense research [35].

Now a new vector, 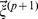 comes to be recorded. Some neurons fire and some don't, accordingly they get values +1 and −1, and through the above *J_ij_*'s, local fields, or PSP's, develop on each neuronal site as,
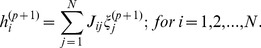
(7)


As explained above the 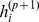
's may or may not match with 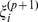
's for all values of *i*, but, in any case, the system would know the difference 
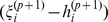
 on each neural site. Note that the computation of this difference on each site already amounts to orthogonalization [7], i.e.
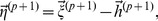
(8)where,
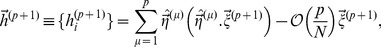
(9)since 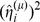 is of the order of 1/*N*.

The interesting new thing we point out here is that if it so happens that 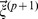 is already in the memory store, say as the 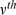 vector (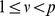), then 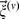 will not project on to 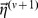,…,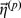 [36], and the first 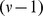 terms in eqn.(9) will give (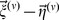). Then,
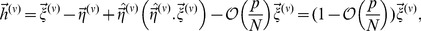
(10)since 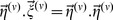. So the presented 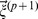 will be identified as 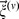, with 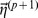 on the order of zero. This would imply that 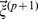 will not be orthogonalized and stored again, no matter how often it is presented. However, if it turns out that 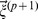 is indeed a new vector, which is not there in the memory store, then 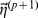 will be computed according to eqn.(8) and will be stored in the synapses following the modified Hebb's learning rule (6). Some clarification is needed here in order to understand how Hebb-Hopfield model with Gram-Schmidt orthogonalization (H-H-G-S) scores over the conventional Hebb-Hopfield (H-H) model.

Let *p* normalized vectors be stored (for *p*/*N* very small, say 0.05) in each of the above two cases, and let a test vector that is similar to (but not exactly the same as) one of the *p* stored vectors be presented to check if it associates with any of the *p* stored vectors. In both the cases the test vector will indeed associate with one of those *p* vectors to which it resembles. This means that in H-H-G-S scheme the *p* imprinted vectors are stable in the same way as in the H-H scheme, i.e. they have non-zero basins of attraction [30], [37], and that the test vector, which falls within the basin of attraction of one of the imprinted vectors, converges to the imprinted vector. Thus the attractor neural network (ANN) character typically attributed to H-H is preserved in H-H-G-S.

To elaborate further we note that two processes are involved in this: (i) ‘storage’ of information (or vectors) in the synapses through eqns. (2) and (6) respectively in the two cases; and (ii) ‘association’ of a presented test vector with one of the memorised vectors through prescriptions (3) and (7) respectively. The two processes are invoked independently in H-H in that when a new vector is presented we have to specify whether the process of ‘storage’ needs to be invoked or whether the vector is meant to be ‘associated’ with a vector in the memory. If it is instructed to be stored then it will be stored regardless of the extent of its similarity or difference with any of the vectors already in the memory. But in H-H-G-S the two processes are linked.

When a new vector is presented to the H-H-G-S scheme for storage, it has to be first orthogonalized, and as part of orthogonalization it is first subjected to a check, through eqn.(7), whether it ‘associates’ with any of the stored vectors, and if so, with which one. If it falls within the basin of attraction of one of the stored vectors [30] then it will be associated with that particular vector in the memory store and signs of 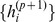 will coincide with those of the components of that vector. In case the new vector is not similar to any of the stored vectors then 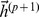 will be an independent vector that holds the information of the overlaps of the new presented vector with all the stored vectors in a convoluted manner.

The above amounts to half of the orthogonalization process. The process is completed with the comparison (through eqn.(8)) of the new presented vector with 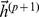, which may correspond either to one of the stored vectors or to a vector very different from any one of them. The difference calculated by eqn.(8) will be small or large depending on the two situations, but in either case this will tantamount to orthogonalization and the orthogonalized version of the new vector will be ‘stored’ according to eqn.(6). In case the presented new vector happens to be identical (not just 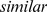) to a vector already in the memory store then, as shown in eqn.(10), 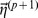 will be identically zero.

The H-H-G-S scheme thus appears to be close to reality in which when the brain encounters a new information, before storing it, it knows, in the background of the information already in its memory, that the new information is completely familiar, or completely unfamiliar, or partially familiar. This is accomplished by the first part of orthogonalization represented by eqn.(7), namely ‘association’.

The crucial implication in the present context of CI is that orthogonalization diminishes the overlap of any pattern that comes to be recorded with everyone of those that are already in the store and thus suppresses the noise 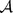. The PSPs, 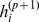
's on all the sites *i*, are pinned at 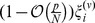. Since 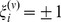, the PSP's are strictly confined within the range 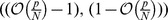. Thus, already familiar stimuli are blocked from stimulating the system again and again to cause overloading and a possible run-away potentiation.

In Figure 2 we present results of our simulations showing (a) how the retrieval quality drops rapidly around *p*/*N* = 0.14 signifying CI, and (b) how Gram-Schmidt orthogonalization overcomes catastrophic interference. We use a system comprising 1000 neurons. Patterns are generated using pseudo-random number generators to assign values +1 and −1 to the neurons. The patterns are learnt sequentially and stored by changing the synaptic efficacy *J_ij_* and accumulating the changes as in eqn.(2). Soon after a pattern is stored, it is presented back to the network to check if it can be retrieved using the prescription elaborated in eqns.(3–5). Figure 2(A) shows the fraction of retrieval, i.e. the ratio, (no. of retrieved patterns)/(no. of learnt patterns), versus load parameter, which is the ratio of (no. of learnt patterns)/(total number of neurons), i.e. *p*/*N*. Around *p*/*N* = 0.14 the fraction of retrieved patterns dips below 90% quite rapidly and reduces to almost zero around *p*/*N* = 0.17. The results are shown for three sets of input patters. The inset shows the same plot after averaging over 18 sets of patterns. Figure 2(B) shows the same calculation after invoking Gram-Schmidt orthogonalization on the incoming patterns – an incoming pattern is first orthogonalized with respect to all the stored patterns (using eqn.(8)) and then stored, but the original, or the raw pattern (before orthogonalization) is tested for retrieval. In a system of 1000 neurons all presented patterns are retrieved perfectly until *p* = 998. For *p* = 999 the fraction of retrieved patterns dips abruptly to almost zero, and to exactly zero when *p* = 1000 as amplified in the inset.

Even though by storing orthogonalized patterns the memory capacity appears to rise from 0.14*N* to almost *N* it is important that we check the stability of the stored memories. As stated above we should do it by computing the basins of attraction for the memories. Using the standard definitions [30], [37] we did the simulations for a smaller network of 100 neurons to get an idea as to how the size of basin of attraction changes when we introduce orthogonalization.

To get the right perspective we first did the calculations for the conventional Hopfield model. The network was made to learn 12 randomly generated 100-dimensional patterns (of +1 and −1) according to eqn.(2). The patterns were then picked up one by one and states of certain neurons were switched (from −1 to +1 or vice versa) – starting with switching of state of one neuron chosen randomly – and it was checked if the chosen imprinted pattern, say 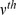, could be retrieved following the prescription of eqn.(5). If the signs of 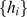 did not match with those of the imprinted 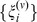 then 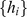 were fed to the right hand side of eqn.(5) as 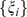 and new 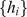 were calculated and their signs were compared with those of the imprinted 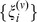. A maximum of 10 such iterations were tried to check if they led to convergence to the imprinted 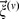. This exercise was repeated for 10 samples generated by picking the ‘flipped’ neuron from 10 different locations chosen randomly in the array of 100 neurons.

The above procedure was repeated by switching signs of more and more neurons successively until the overlap of the retrieved pattern with the corresponding imprinted pattern fell below 100%. This marked the size of basin of attraction for a particular imprinted pattern.

For the conventional Hopfield model the basin of attraction for 12 imprinted patterns were distributed in a broad range from 26 to 44, with maximum probability for basins of sizes 34 to 37. As the number of imprinted patterns increased beyond 10 certain patterns began to show absence of basin of attraction (i.e. basin of size zero). Beyond 14 memorised patterns the number of patterns with zero basin of attraction increased rapidly.

Orthogonalization improves the situation considerably. We considered the same 12 patterns but stored their orthogonalized versions. The original patterns (before orthogonalization) were considered for retrieval and basins of attraction were computed for them. The sizes of basins ranged between 6 and 45 but were concentrated around 31. From *p* = 14 certain patterns begin to lose basin of attraction (i.e. basin of attraction of size zero) though with very small probability, about 0.0093. The probability increases quite rapidly with *p*, becoming 0.49 at *p* = 24 and 1.0 when *p* touches 100. Thus orthogonalization presents an interesting scenario in which in a system of *N* neurons up to (*N*−1) patterns are stored and retrieved efficiently, and therefore compete for space for basin of attraction. There are several interesting issues that need close investigation. We are in the process of carrying them out.

## Discussion

Many approaches have been used to try and overcome the problems of the actual or predicted loss of stored information in memory systems, both in connectionist networks (catastrophic interference) and in biological systems (e.g. ongoing plasticity, [4]; the stability-plasticity problem, [1]). A system has to be flexible enough to allow salient changes to be encoded continuously while at the same time being stable enough to ensure that stored changes persist. The approach that we show here uses a conventional Hopfield network. It thus makes no claims to be biologically realistic in the sense that it includes details of neuronal or synaptic physiology, but we feel that this simple case allows us to address fundamental issues of the stability-plasticity dilemma. The approach that we use allows the same components to encode and store information. In fact, rather than try and separate stored and new inputs, the input is instead considered in the context of previously stored inputs, which means that only the similarities and differences of new inputs are encoded while still allowing the full memory of the input to be recalled.

We are able to show the capability of encoding and storing a significantly larger number of sequential inputs than is possible using conventional approaches, and importantly, allowing new inputs to be compared and generalized to those already in the store. This contrasts with the non-overlapping approaches used in connectionist networks in attempts to overcome catastrophic interference (e.g. [38]; see [12]). While separation of input patterns would remove catastrophic interference, it also removes the possibility of generalising and linking together aspects of the stored patterns. This could be a particular problem for learning categories [17]. That a pattern to be stored is compared to those already in the store, without having to impose limits on the rate or extent of the synaptic changes, is a principal advantage of the orthogonalization approach that we show here.

In human memory systems the subject learns on the background of previously stored information rather than isolating the new information from it, or overwriting the previously stored information (see [39]). This feature is an intrinsic component that arises from Gram-Schmidt orthogonalisation rather than having to be imposed from outside. This could allow artificial, and in principle biological systems, to make use of an intrinsic principle of physical systems, ensuring that a system that includes this automatically has this advantage built in. An orthogonalization based neural system acts in a self-organized manner - it compares the new with old, isolates the similarities and differences of the new input with the old, deduces whether the new is unknown or known, and if it is found to be known to it then it refuses to entertain it a second time. In this way it acts as a form of “internal supervisor” [4], determining which synapses have to change to store the new memory while not destroying the changes at synapses that have previously stored information. A stimulus may be presented any number of times but if the input has already been stored then the postsynaptic local field will not change and therefore they will not build up incessantly in the same direction to cause the possible run-away effect, akin to that suggested by the BCM model.

Orthogonalisation has been used previously in attempts to overcome the problems of catastrophic interference in connectionist networks (see, for example, [40]). However, the use of the term orthogonalisation in this context differs to the way that we have used it, where information is represented by a vector and orthogonalization makes the vector of a new information perpendicular to the vectors representing the stored information. Orthogonalized, or mutually perpendicular, vectors do not overlap with each other. This orthogonalization scheme must be distinguished from the ‘orthogonalization’ approach that is typically used in the learning and memory literature (e.g. [41], [22], [6] and references therein). The latter generally refers to sparse coding of information in the network, i.e., two different pieces of information are stored on two non-overlapping sets of nodes in the network, thus removing the interference effect associated with CI. However, in the scheme presented here the same nodes are used. If patterns of bipolar elements are generated randomly, at the first glance they could be considered orthogonal (i.e., with zero inner product). This would be true in the hypothetical situation of infinite systems (when vectors have an infinite number of components). However, since we are always dealing with finite vectors, inputs of this sort will be only approximately orthogonal, and the inner products will be non-zero. This is not orthogonalization by design, and the non-zero overlaps mean that the signal gets submerged in the noise when *p*/*N*>0.14 [42]. The typical/common notion of orthogonal patterns is, thus sparsely coded non-overlapping patterns (see also [43]), and by whatever means it is achieved this can help reduce CI (see [40]). The Gram-Schmidt orthogonalization that we use differs as it forces the network to actively compute and convert a set of vectors into a mutually orthogonal set. In this process the noise arising due to the intrinsic overlap amongst patterns, even though they are generated randomly, is eliminated and the memory capacity increases to *p*/*N* = 1 from 0.14.

We have examined an artificial system, and the relevance of this effect ideally needs to be shown in an experimental system. While we, and others, believe that the approach can say something relevant to actual systems, this needs to be tested as even in theoretical systems effects differ as the degree of realism changes (see [18]). That there are sliding thresholds for plasticity is known from experimental analyses (see [27]), but that inputs can be orthogonalised requires certain network arrangements and cellular conditions for its implementation. These include parallel feedforward excitation and feedback inhibition [42], as well as the nature of inputs to single and different dendrites of the same cell, and multiplication in dendrites (see [43]). All of the constraints needed are common network motifs or identified functional properties in biological systems, offering the possibility of testing these predictions experimentally.

## References

[1] Abraham WC, Robins A (2005) Memory retention–the synaptic stability versus plasticity dilemma. Trends Neurosci. Feb 28(2): 73–8. 10.1016/j.tins.2004.12.003 15667929

[2] Bienenstock E, Cooper L, Munro P (1982) Theory for the development of neuron selectivity: orientation specificity and binocular interaction in visual cortex. J Neurosci 2: 32–48. 7054394 10.1523/JNEUROSCI.02-01-00032.1982 PMC6564292

[3] Bliss T, Collingridge G (1993) A synaptic model of memory: long-term potentiation in the hippocampus. Nature 361: 31–39. 8421494 10.1038/361031a0

[4] Fusi S, Abbott LF (2007) Limits on the memory storage capacity of bounded synapses. Nat Neurosci. Apr 10(4): 485–93. 10.1038/nn1859 17351638

[5] Fiskum G (2000) Mitochondrial participation in ischemic and traumatic neural cell death. J Neurotrauma 17: 843–855. 11063052 10.1089/neu.2000.17.843

[6] French R (2003) Catastrophic interference in connectionist networks. Encyclopedia of Cognitive Science 1: 431–435.

[7] Srivastava V, Edwards SF (2000) A model of how the brain discriminates and categorises. Physica A 276: 352–358.

[8] Srivastava V, Edwards SF (2004) A mathematical model of capacious and efficient memory that survives trauma. Physica A 333: 465–477.

[9] Nader K, Schafe G, Doux JL (2000) Fear memories require protein synthesis in the amygdala for reconsolidation after retrieval. Nature 406: 722–726. 10963596 10.1038/35021052

[10] Ben Mamou C, Gamache K, Nader K (2006) NMDA receptors are critical for unleashing consolidated auditory fear memories. Nature Neuroscience 9(10): 1237–1239. 16998481 10.1038/nn1778

[11] Barnes JM, Underwood BJ (1959) Fate of first-list associations in transfer theory. Journal of Experimental Psychology 58: 97–105. 13796886 10.1037/h0047507

[12] French R (1999) Catastrophic forgetting in connectionist networks. Trends Cogn Sci 3: 128–135; Guyon I, Personnaz L and Dryfus G (1989) Of points and loops. NATO ASI Series, F41, Eckmiller R and Malsburg Ch v d (Eds): 261–269. 10.1016/s1364-6613(99)01294-2 10322466

[13] Ratcliff R (1990) Connectionist Models of Recognition Memory: Constraints Imposed by Learning and Forgetting Functions. Psychological Review 97: 285–308. 2186426 10.1037/0033-295x.97.2.285

[14] Shadmehr R, Brashers-Krug T (1997) Functional stages in the formation of human long-term motor memory. The Journal of Neuroscience 17 (1): 409–419. 8987766 10.1523/JNEUROSCI.17-01-00409.1997 PMC6793707

[15] Mareschal D, Quinn PC, French RM (2002) Asymmetric interference in 3-to-4 months olds' sequential category learning. Cognitive Science 26: 377–389.

[16] Fusi S, Dre PJ, Abbott LF (2005) Cascade models of Synaptically Stored Memories. Neuron 45: 599–611. 15721245 10.1016/j.neuron.2005.02.001

[17] McClelland JL, McNaughton BL, O'Reilly RC (1995) Why there are complimentary learning systems in the hippocampus and neocortex: insights from successes and failures of connectionist models of learning and memory. Psychological Review 102 (3): 419–457. 7624455 10.1037/0033-295X.102.3.419

[18] Fusi S, Senn W (2006) Eluding oblivion with smart stochastic selection of synaptic updates. Chaos 16: 026112. 16822044 10.1063/1.2213587

[19] Kempermann G (2008) The neurogenic reserve hypothesis: what is adult hippocampal neurogenesis good for? Trends Neurosci 31: 163–169. 18329110 10.1016/j.tins.2008.01.002

[20] Bogacz R, Brown M (2003) Comparison of computational models of familiarity discrimination in the perirhinal cortex. Hippocampus 13: 494–524. 12836918 10.1002/hipo.10093

[21] van Rossum M, Shippi M, Barrett A (2012) Soft-bound Synaptic Plasticity Increases Storage Capacity. PLoS Comput Biol 8: e1002836. 23284281 10.1371/journal.pcbi.1002836 PMC3527223

[22] French R (1997) Selective memory loss in aphasics: An insight from pseudo-recurrent connectionist networks. In: Connectionist Representations. (Bullinaria J, Glasspool D, Houghton G, eds), pp 183–195: Springer.

[23] McCloskey M, Cohen N (1989) Catastrophic interference in connectionist networks: the sequential learning problem, in The Psychology of Learning and Motivation (Vol. 24 ) (Bower, G.H., ed.), pp. 109–164, Academic Press.

[24] Bear M (2003) Bidirectional synaptic plasticity: from theory to reality. Phil Trans R Soc Lond B 358: 649–655. 12740110 10.1098/rstb.2002.1255 PMC1693164

[25] Turrigiano G (2007) Homeostatic signaling: the positive side of negative feedback. Current Opinion in Neurobiology 17: 318–324. 17451937 10.1016/j.conb.2007.04.004

[26] Bear M (1996) A synaptic basis for memory storage in the cerebral cortex. Proc Natl Acad Sci 93: 13453–13459. 8942956 10.1073/pnas.93.24.13453 PMC33630

[27] Abraham W, Bear M (1996) Metaplasticity: the plasticity of synaptic plasticity. TINS 19: 126–130. 8658594 10.1016/s0166-2236(96)80018-x

[28] Representation of a network state as a vector of +/−1 as opposed to 0/1 has a distinct mathematical advantage. The central one is that the overlap between two states, or vectors, can be represented by the dot (or scalar) product of the two vectors. See [30] for a comparison between these two alternatives, though equivalent, notations for the neural states.

[29] Cooper L (1973) A possible organization of animal memory and learning, in Nobel Symposium on Collective Properties of Physical Systems. The Nobel Foundation: Aspensagaerden, Sweden:: 62–84.

[30] Amit D (1989) Modeling Brain Function: The world of attractor neural networks. Cambridge University Press; Dayan P and Abbott L F (2001) Theoretical Neuroscience. MIT Press, Cambridge, Massachusetts.

[31] Hebb D (1949) The organization of behavior: A neuropsychological theory. Wiley, New York.

[32] Bi G-Q, Poo M-M (1998) Synaptic modifications in cultured hippocampal neurons: dependence on spike timing, synaptic strength, and postsynaptic cell type. J Neurosci 18: 10464–10472. 9852584 10.1523/JNEUROSCI.18-24-10464.1998 PMC6793365

[33] Miller K (1996) Synaptic Economics: Competition and Cooperation in Synaptic Plasticity. Neuron 17: 371–374. 8816700 10.1016/s0896-6273(00)80169-5

[34] Srivastava V, Vipin M, Granato E (1998) Recall of Old and Recent Information. Network Comput. Neural Syst 9: 159–166. 9861983

[35] Carandini M, Heeger DJ (2012) Normalization as a canonical neural computation. Nature Reviews Neuroscience 13: 51–62. 10.1038/nrn3136 PMC3273486 22108672

[36] Srivastava V (2000) A unified view of the orthogonalization methods. J Phys A: Math Gen 33(35): 6219–6222.

[37] Bar-Yam Y (1997) Dynamics of Complex Systems. Addison-Wesley, Massachusetts.

[38] Kruschke JK (1992) ALCOVE: an exemplar-based model of category learning. Psychological Review 99 (1): 22–44. 1546117 10.1037/0033-295x.99.1.22

[39] McRae K, Hetherington PA (1993) Catastrophic interference is eliminated in pretrained networks. In Proceedings of the 15th Annual Conference Sciences Society (Hillsdale NJ: L. Erlbaum), pp. 723–728.

[40] Yamaguchi M (2004) Reassessment of catastrophic interference. Neuroreport 25: 2423–2426. 10.1097/00001756-200410250-00024 15640768

[41] Lewandowsky S, Li S-C (1993) Catastrophic interference in neural networks: causes, solutions, and data. In: New Perspectives on Interference and Inhibition in Cognition (Dempster F, Brainerd C, eds), p 329–361: Academic Press.

[42] Srivastava V, Parker D, Edwards SF (2008) The nervous system might ‘orthogonalize’ to discriminate. J Theor Biol 253: 514–517. 18511085 10.1016/j.jtbi.2008.03.031

[43] Marr D (1969) A theory of cerebellar cortex. J Physiol 202: 437–470. 5784296 10.1113/jphysiol.1969.sp008820 PMC1351491

